# Seven hundred years of human-driven and climate-influenced fire activity in a British Columbia coastal temperate rainforest

**DOI:** 10.1098/rsos.160608

**Published:** 2016-10-26

**Authors:** Kira M. Hoffman, Daniel G. Gavin, Brian M. Starzomski

**Affiliations:** 1 School of Environmental Studies, University of Victoria, 3800 Finnerty Road, Victoria, British Columbia, CanadaV8P 5C2; 2 Hakai Institute, Calvert Island, PO Box 309, Heriot Bay, British Columbia, CanadaV0P 1H0; 3 Department of Geography, 1251 University of Oregon, Eugene, OR 97403-1251, USA

**Keywords:** anthropogenic burning, Arctic Oscillation, coastal temperate rainforest, El Niño-Southern Oscillation, Pacific Decadal Oscillation, pacific northwest

## Abstract

While wildland fire is globally most common at the savannah-grassland ecotone, there is little evidence of fire in coastal temperate rainforests. We reconstructed fire activity with a *ca* 700-year fire history derived from fire scars and stand establishment from 30 sites in a very wet (up to 4000 mm annual precipitation) temperate rainforest in coastal British Columbia, Canada. Drought and warmer temperatures in the year prior were positively associated with fire events though there was little coherence of climate indices on the years of fires. At the decadal scale, fires were more likely to occur after positive El Niño-Southern Oscillation and Pacific Decadal Oscillation phases and exhibited 30-year periods of synchrony with the negative phase of the Arctic Oscillation. Fire frequency was significantly inversely correlated with the distance from former Indigenous habitation sites and fires ceased following cultural disorganization caused by disease and other European impacts in the late nineteenth century. Indigenous people were likely to have been the primary ignition source in this and many coastal temperate rainforest settings. These data are directly relevant to contemporary forest management and discredit the myth of coastal temperate rainforests as pristine landscapes.

## 1. Introduction

Palaeofire studies have found evidence that both humans and climate influence fire regimes, but it is often difficult to discern their relative importance, and the drivers of fire and its ecosystem effects remain poorly understood [1,2]. Humans affect fire regimes by controlling ignition and suppression, and through altering vegetation and the availability of fuel types by practices such as land clearing and habitat modification [2–5]. Climate drives fire activity through interactions with local-scale, bottom-up controls such as available fuels and topography to affect fuel drying and fire spread and by influencing rates of natural ignitions [1,6,7]. In the Pacific Northwest (PNW) of North America, it is often difficult to attribute historic drivers of fire activity and causes of fire ignitions due to both the abundance of natural ignitions from lightning and the impact of logging, grazing and fire suppression that decreased fire occurrence soon after the loss of Indigenous fire management [1–3].

Indigenous peoples used fire for diverse purposes such as clearing land, creating fodder for game, and increasing the productivity of specific plants for food and medicine [8,9]. The cultural importance of fire has been mostly examined in the savannah-grassland ecotone [2,3,8] and in fire-adapted forests dominated by ponderosa pine (*Pinus ponderosa*) and Garry oak (*Quercus garryana*) [10–12]. There has been very little documented evidence of historic fire activity in coastal temperate rainforests located in the PNW and previous disturbance studies have asserted that both human- and lightning-caused fires are infrequent and become progressively rarer in high-latitude regions (more than 50° N) [13–16]. Low fire activity is assumed due to the presence of old growth forests [14], the lack of fire adaptations in dominant conifer species [13], the decreased flammability of large-diameter, moisture-laden surface fuels [1,3] and the absence of twentieth-century fire activity [17]. There have been no reconstructions of historic fire activity with fire-scarred trees in these wet and dense forests, and fires are hypothesized to be large, stand-replacing events occurring during unusually dry years when low fuel moisture promotes rapid fire spread [2,13]. A handful of studies in coastal temperate rainforests of British Columbia have described temporally coarse reconstructions of Holocene fire activity with charcoal stratigraphy in lake sediments [18] and radiocarbon dating of soil charcoal [19–21]. These studies depict patchy fires during the last few centuries embedded within landscapes experiencing long (sometimes more than 1000 year) fire intervals as well as widespread increases in fire activity in the Mid- and Late Holocene [19–21] that may be related to increases in the use of fire by Indigenous peoples [21].

Wildfire occurrence is also influenced by variability in modes of sea surface temperatures (SSTs) such as the El Niño-Southern Oscillation (ENSO) and the Pacific Decadal Oscillation (PDO) that influence winter temperature and precipitation patterns and indirectly affect summer moisture availability [22–24]. The ENSO is characterized by oscillating phases of warmer (El Niño) and cooler (La Niña) than average winter SSTs in the equatorial Pacific at interannual (2–7 year) frequencies [25]. The ENSO affects climate and fire in distinct ways depending on ocean–atmosphere conditions and their associated circulation patterns [24]. The warm (El Niño) phase produces warmer and drier winter and spring conditions in the PNW, which enhances wildfire activity in summer months [23,24]. The PDO is characterized by variations in SSTs in the North Pacific and positive (negative) PDO values produce relatively similar climate and circulation patterns to El Niño (La Niña) conditions though the periodicity is decadal (approx. 20 years) [26].

The Arctic Oscillation (AO) is the dominant mode of sea-level pressure (SLP) variation north of 20° N and is similar to ENSO in its interannual periodicity [27]. Variability in SLP has been shown to indirectly affect fire activity in high-latitude regions by affecting the position of the jet stream [28]. It is characterized in its positive (negative) phase by negative (positive) pressure anomalies that form over the Arctic and a circumpolar belt of positive (negative) temperature anomalies at mid-latitudes, with the exception of low-temperature anomalies that form over the PNW [27–29]. In Western Canada and Southeast Alaska, large fire incidents have been linked to the negative phase of the AO when high-pressure anomalies centred over the Arctic extend south to 30–50° N and result in warmer and drier conditions that are more conducive to fire initiation and spread [29]. Only a few studies have assessed fire–climate relationships between the ENSO, PDO and AO in British Columbia. These studies have omitted coastal temperate rainforests from their analyses, suggesting that these forests are too wet to show summer moisture deficits and are therefore unable to support wildfires [17,30].

In this study, we overcome the limitations of past studies by reconstructing the fire history of a coastal island with extremely low rates of lightning and a short summer dry season. Indigenous peoples abandoned the island more than 100 years ago, and it never had Euro-American settlement, logging, grazing or fire suppression [31,32]. We hypothesize that if Indigenous people frequently used fire, the fire history record would be temporally and spatially associated with former habitation sites at decadal intervals and absent in the twentieth century. If Indigenous people rarely used fire, fire would be consistently rare, occurring at centennial and millennial intervals and mostly explained by topographic and climatic controls. We evaluate the relationship between climate variability and humans on fire occurrence over *ca* 700 years in a high-latitude coastal temperate rainforest. We use a network of fire scars and stand establishment to address the following questions: (i) What is the relationship between the temporal occurrence of fire events with drought (The Palmer Drought Severity Index (PDSI)) and with single and interacting phases of climate drivers such as the ENSO, PDO and AO? (ii) Is fire occurrence best explained by human presence, regional climate variability, topography or more likely, interactions among these drivers?

## 2. Material and methods

### 2.1. Study area

**Figure 1.**
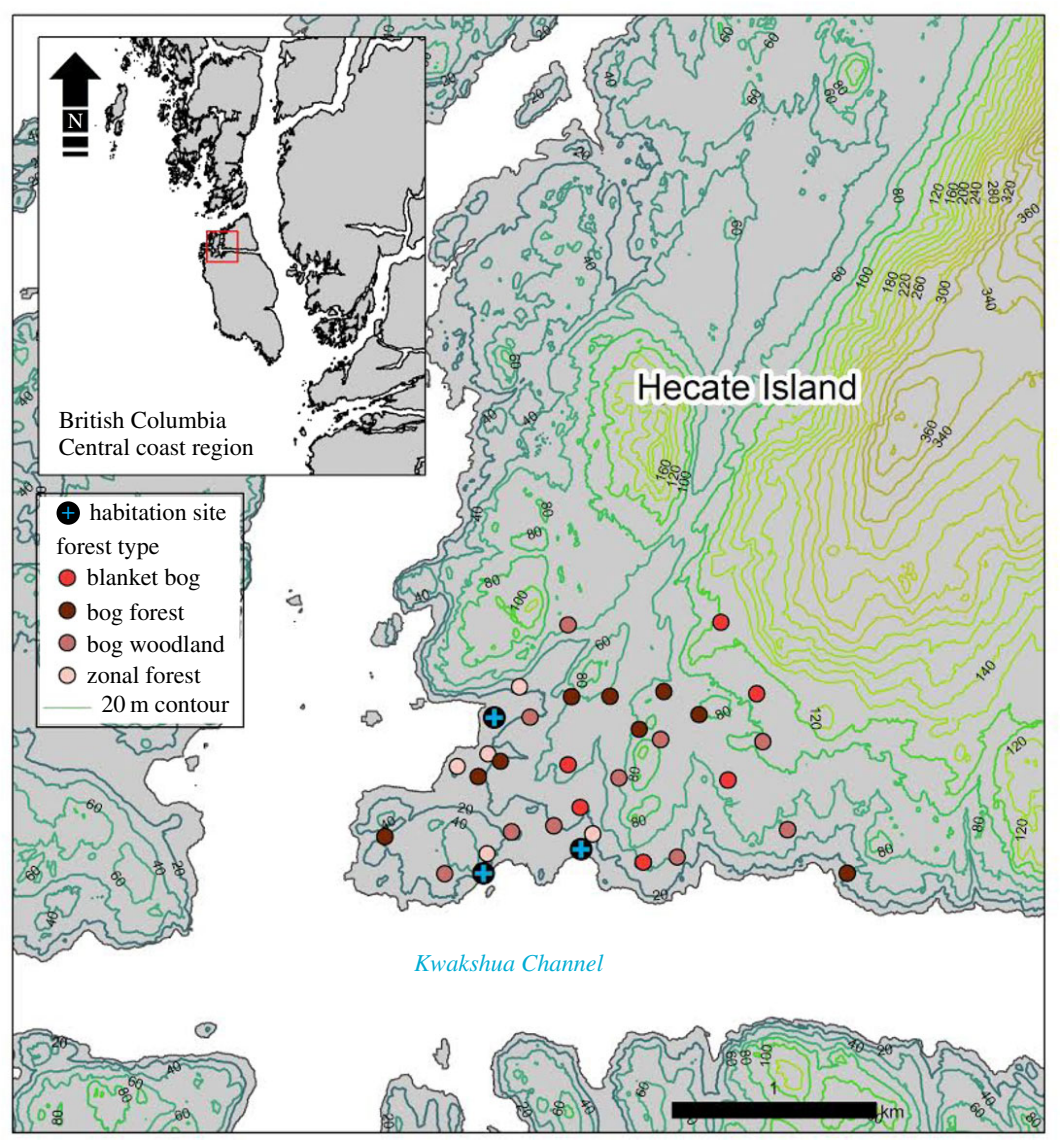
The locations of sample sites on Hecate Island (latitude 51°39′43 N and longitude 128°04′2 W). The shaded circles (four vegetation types) represent the locations of 30 fire history plots and the blue crosses provide the general location of the three former habitation sites in the study area. The inset provides the location of the study site on the central coast of British Columbia, Canada.

The study area is located on Hecate Island (latitude 51° 39′ N, longitude 128° 04′ W) within the Hakai Lúxvbálís Conservancy on the central coast of British Columbia, Canada (figure 1). This region is characterized by a temperate climate with year-round cool temperatures (average annual approx. 7°C, average summer approx. 12°C) and annual rainfall sometimes exceeding 4000 mm [33]. Hecate Island (67 km^2^) is located in the very wet hypermaritime subzone of the Coastal Western Hemlock biogeoclimatic classification [34]. Excess soil water regulates this environment and subtle variations in slope or drainage result in significant differences in forest productivity [33]. Four general vegetation types defined by dominant species and closely associated landforms are found in the study area [33]. Productive (zonal) forests found in near shore and riparian areas are characterized by large-diameter western red-cedar (*Thuja plicata* Donn ex D. Don.) and western hemlock (*Tsuga heterophylla* (Raf.) Sarg.), with lesser amounts of yellow-cedar (*Cupressus nootkatensis* (D. Don) Farjon & Harder) and Sitka spruce (*Picea sitchensis* (Bong.) Carr.) [34]. Bog forests exhibit stunted growth forms and are located on hill slopes dominated by western red-cedar, yellow-cedar, western hemlock and shore pine (*Pinus contorta var. contorta* Douglas ex Louden) [35]. Bog woodlands are the most common vegetation type and comprise patchy mosaics of forested and unforested sites in subdued or rolling terrain [33]. These forests contain roughly equal densities of western red-cedar, yellow-cedar and shore pine with lesser amounts of mountain hemlock (*Tsuga mertensiana* (Bong.)) [35]. Blanket bogs are nutrient-poor, sparsely forested wetland areas that contain small amounts of shore pine and yellow-cedar [33]. Zonal and bog forest vegetation types have closed canopies with larger diameter and more vertical fuel structures that are blanketed by thick moss compared with bog woodland and blanket bog vegetation types, which are more open, drier and contain finer, more flammable fuel assemblages [14,21]. Elevations in the study area range from sea level to 150 m, and the geological substrate is homogeneous quartz diorite and granodiorite [36].

Although there are no oral histories of fire use by Indigenous people on Hecate Island, Indigenous peoples continuously inhabited the study area for at least 13 000 years until European contact in the late eighteenth and nineteenth centuries [6,7]. A team of archaeologists used radiocarbon dating of shell, faunal and charcoal deposits to determine the locations of former habitation sites [31,32]. This evidence together with the presence of culturally modified trees and near shore features, including shell middens, clam gardens and fish traps confirm that Hecate Island supported three year-round habitation sites that were consistently used throughout the Holocene [31,32] and established millennia prior to the rise of contemporary vegetation in the region [37]. Although Indigenous groups continue to use the study area for seasonal resource gathering, habitation sites have not been occupied since the late nineteenth century coinciding with smallpox epidemics and the relocation of people to government-imposed reservations [37–39].

### 2.2. Fire history sampling design

To reconstruct a *ca* 700-year fire history, we sampled fire scar and stand establishment dates from 30 plots (11.28 m radius (0.04 ha)) selected using a stratified random sampling design representing the range of elevations, aspects and vegetation types within a 300 ha study area spanning three watersheds on Hecate Island (figure 1). To reconstruct low and mixed severity fires, we used a chainsaw to remove partial wedge sections of fire scars from the bases of the oldest sound living trees (determined after coring and measuring) [40]. Fire-scarred trees were sampled in the 1 ha area surrounding every plot. We validated the year of each fire event recorded on fire scar wedges by sampling two 5 mm increment cores roughly 1.3 m from the ground from approximately 300 trees which were collected outside the fire area to ensure cross-dating accuracy in the fire scar chronology [41]. In every plot, we recorded diameter at breast height (DBH) and removed two 5 mm increment cores from the root collar of every living tree more than 7.5 cm DBH to define the ages of post-fire cohorts within each plot and to estimate the year of the fire in which the cohort established [41,42]. Because fire scars provide more accurate records of fire occurrence, we assigned post-fire cohorts to the same year as nearby fire events if they occurred within the 10-year period of a nearby fire scar [42].

In the laboratory, cores and wedges were processed using standard dendrochronological techniques [43]. Samples were first visually cross-dated and then statistically verified using the computer program COFECHA [44]. We cross-dated fire scars to species-specific chronologies to obtain exact fire years (the calendar year in which the scar formed) [7]. We were unable to determine the intra-ring position (seasonality) of the majority of samples due to rot and narrowing of tree rings at fire scar margins [45]. We determined the fire history in each plot by compiling fire scars into a composite chronology, and identified fire events as those in which at least two trees had fire scars [41]. Composite fire histories were graphed using Fire History Analysis and Exploration Software [46].

### 2.3. Spatial controls of the fire regime

We used a generalized linear model (GLM) with a Poisson distribution and model selection via the Akaike information criterion (AIC_c_) to predict two response variables, (i) the abundance of fire scars and (ii) the frequency of fire events, using the explanatory variables (vegetation type, slope, aspect, elevation, distance to shoreline and distance to former habitation site) in the ‘MuMIn’ package in R statistical software [47,48] following the methods of Harrell [49] and Burnham & Anderson [50]. Because there were no recorded fire events in the study area after 1893, we only calculated the mean fire interval (MFI) from the first detected fire in 1376 to the last detected fire in 1893 (517 years), and we assessed the composite MFI by combining all fire scar data from the study area over this period of analysis [41]. We were able to estimate fire effects on vegetation (severity) using the presence of fire-scarred trees, the MFI and whether cohorts had established after fire events (indicative of mixed severity fires) [45].

### 2.4. Climate datasets

The PDSI is a composite monthly index of regional climate calculated from current and archived instrumental data of precipitation and temperature [51]. Negative PDSI values indicate dry years and positive values indicate wet years (range −6 to +6). To characterize historic patterns of drought in the study area, we used an extended reconstruction of the PDSI from the gridded North American Drought Atlas (grid point 17 (127.5° W × 52.5° N)), which combines 28 tree-ring reconstructions of summer (June–August) temperature and precipitation [52]. In our analyses, we used previous reconstructions of climate variability (PDSI, ENSO, PDO and AO), which correlated to instrumental records and tree-ring chronologies sampled from the study area (electronic supplementary material, table S1).

### 2.5. Annual fire–climate analyses

At the annual scale, frequency analysis was evaluated with a G-test of goodness of fit in the ‘DescTools’ package in R statistical software [48,53] to determine whether fire events were more likely to occur during extreme climate years of single climatic indexes (negative and positive phases of the PDSI, ENSO, PDO and AO). We used this test again to consider the influence of two- and three-way interactions between the ENSO, PDO and AO with fire occurrence [54]. We compared the number of fires during phase combinations assuming an equal likelihood of burning in all years. Sixty per cent of fire events occurred between 1700 and 1893, therefore, we chose to analyse three-way interacting climate indexes during this period and compared all two-way interactions using their respective temporal windows (e.g. ENSO 1300–2004 with PDSI 1240--2006 (common period of analysis 1300–1893); electronic supplementary material, figure S1).

### 2.6. Interannual and multidecadal fire–climate analyses

To characterize interannual and multidecadal relationships between fire occurrences and the PDSI, ENSO, PDO and AO, we used bivariate event analysis (BEA) with K1D software [55]. BEA is a temporal variant of spatial point pattern analysis, which employs Ripley's *K* function to examine one-dimensional time-series data [56]. This method is advantageous when testing time-series data that are subject to serial autocorrelation and BEA assesses the response of fire years (synchrony, independence or asynchrony) to climate events within a range of temporal windows. Monte Carlo simulations with 1000 replicates were used to randomize climate time series with 95% and 99% confidence envelopes [57]. BEA, as implemented here, assumes a one-directional process where fire events only respond to previous and current climate events [58].

We first tested single climatic indexes and fire years by defining extreme climate events as the 50 most positive or negative annual values of the PDSI, ENSO, PDO and AO [54]. We then tested associations between two- and three-way combinations among the phases of the ENSO, PDO and AO, and we defined extreme climate interactions by combining the 100 highest or lowest ranked annual values [54].

## 3. Results

### 3.1. Fire history

**Figure 2.**
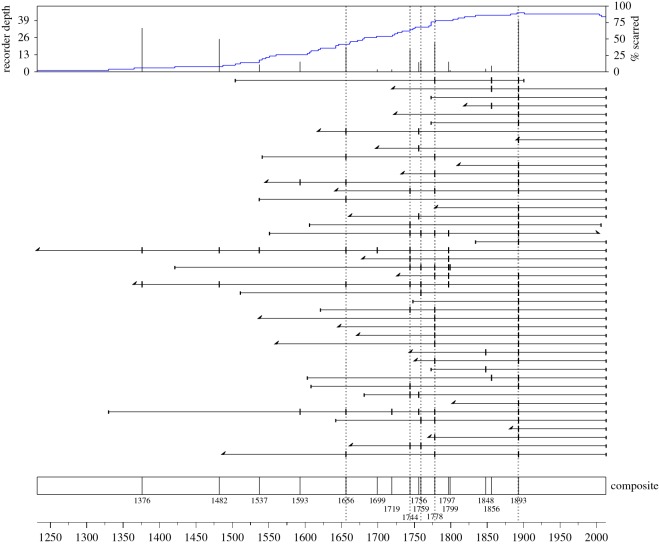
The composite fire history chronology of all fire scars in the study area. Each horizontal line shows the composite fire scar record at a single sampling plot through time. Vertical bars mark fire years and dashed vertical lines mark fire events exceeding 10 ha in size. Fine vertical lines at the beginning and end of each chronology mark pith or bark dates, while fine diagonal lines represent the earliest or latest ring dates for plots where pith or bark dates were not sampled. The sample recorder depth of the chronology is located on the top panel and all fire dates in the study area are recorded in the chronology in the bottom panel.

We detected 13 fire events and three additional fires (recorded with one fire-scarred tree and verified with post-fire cohorts) from 99 fire scars on 45 partial sections of living, fire-scarred trees (figure 2). The majority of partial sections were from western red-cedar (73%) and we found an average of two fire scars per sample (maximum 7) and 27% of trees had only one scar. We cored over 3000 trees (age range 52–953 years) and sampled 9–92 trees (mean = 52 trees) from each of 30 plots (electronic supplementary material, table S2). Our fire evidence was derived from fire scars and confirmed with stand establishment data, which reliably extended to 1325 (689 years, sample depth more than five trees). The year of maximum fire synchrony and the last detected fire event in the study area was in 1893, when 90% of plots burned in a 287 ha mixed severity fire, in which 66% of trees survived (figure 2). The point fire interval (PFI (the MFI at the tree scale)) was 95 years and the composite MFI was 39 years in the period 1376–1893 (electronic supplementary material, table S2).

### 3.2. Controls on the spatial variation of fires

**Figure 3.**
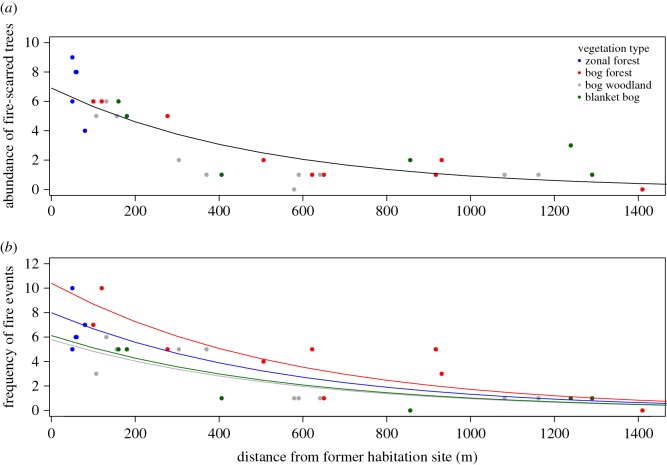
(*a*) Poisson regression of the relationship between the abundance of fire-scarred trees and the distance from habitation sites in metres. The most parsimonious model related the presence of fire scars to distance from habitation sites only, and model averaging indicated that the distance to habitation sites was significantly (*p* < 0.01) more influential than any other predictor or combination of predictor variables. (*b*) Poisson regression of the relationship between the frequency (number) of fire events in each vegetation type and the distance from habitation sites in metres. The most parsimonious model includes three variables (distance to habitation site, aspect and bog forest vegetation type), and distance to habitation site was the most important predictor variable (*p* < 0.001).

The abundance of fire-scarred trees was found to consistently decrease with distance from former habitation sites (GLM Poisson regression: *b* = −1.810 × 10^−3^, *z* = −2.677, *p* < 0.01; figure 3*a*). The most parsimonious model related the abundance of fire scars to distance from former habitation sites only (on the basis of AIC_c_), and model averaging indicated that distance to former habitation site was significantly more influential than any other predictor or combination of predictor variables. Model validation confirmed that distance to former habitation site explained 65% of the variation (*pseudo R*^2^) in the abundance of fire-scarred trees (electronic supplementary material, table S3 and table 1*a*). The second response variable (frequency of fire events) modelled with a Poisson GLM confirmed that the number of fire events in plots decreased with distance from former habitation sites (GLM Poisson regression: *b* = −0.0016, *z* = −4.750, *p* < 0.0001; figure 3*b*). The most parsimonious model related the frequency of fire events to three main predictors (distance from former habitation site, aspect and bog forest vegetation type; on the basis of AIC_c_; table 1*b*). Model validation confirmed that distance to former habitation site was the most important predictor and together with aspect and bog forest vegetation type explained 52% of the variation in the frequency of fire events (table 1*b* and electronic supplementary material, table S4).

**Table 1.** Results of the final model selection using Akaike Information Criterion (AIC_c_) for 11 GLMs that describe (*a*) the abundance of fire-scarred trees and (*b*) the frequency of fire events with nine predictor variables (vegetation (four types), elevation, slope, aspect, distance to habitation site in metres and distance to shoreline in metres). Full results are provided in the electronic supplementary material, tables S3 and S4. Model averaging was conducted and only models within 95% confidence intervals are included. *K* = number of model parameters, *R*^2^ = the pseudo *R*^2^-value, ΔAIC_c_ = change in AIC score from the top model, *w_i_* = AIC_c_ model weight, ER = top model weight divided by *i* model weight.

model	*K*	*R*^2^	ΔAIC_c_	*w_i_*	ER	parameters
(*a*) abundance of fire-scarred trees						
1	1	0.65	0.00	0.42	1.00	distance to habitation
2	2		1.77	0.17	2.53	distance to habitation, slope
3	2		2.02	0.15	2.80	distance to habitation, elevation
4	2		2.39	0.13	3.23	distance to habitation, aspect
(*b*) frequency of fire events						
1	3	0.59	0.00	0.30	1.00	distance to habitation, aspect, bog forest type
2	2		0.75	0.20	1.50	distance to habitation, aspect
3	1		2.31	0.09	3.33	distance to habitation
4	5		2.53	0.08	3.75	distance to habitation, aspect, bog forest type, zonal forest type, distance to shoreline
5	3		2.67	0.08	3.75	distance to habitation, elevation, slope, distance to shoreline
6	4		2.76	0.07	4.28	distance to habitation, bog forest type, zonal forest type, slope
7	3		3.58	0.05	6.00	distance to habitation, elevation, bog forest type

### 3.3. Fire occurrence and single climate indexes

**Figure 4.**
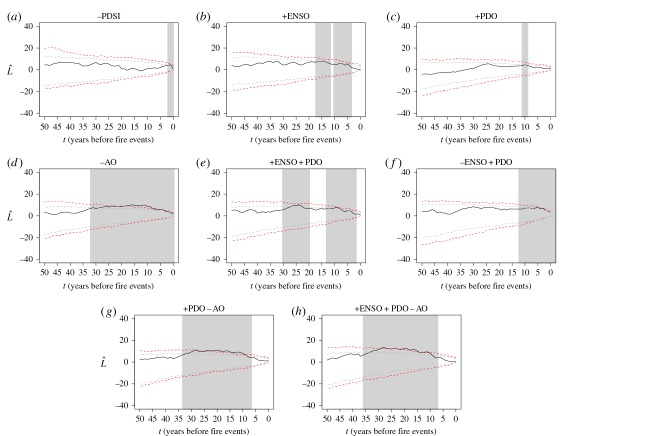
Bivariate event analysis (BEA) of the temporal association between fire years and extreme climate events. Abbreviations are the ENSO, the PDO, the PDSI and the AO, and the (+) and (−) signs indicate phase combinations. Black lines above the dotted red (99% confidence envelopes) and the dotted grey (95% confidence envelopes) lines indicate synchrony between the two events (events occurred more often than expected, *t* years prior to fire events). Confidence envelopes are based on 1000 Monte Carlo simulations and years of significant synchrony are shaded in grey. Further results are provided in the electronic supplementary material, table S5, figures S2 and S3.

Frequency analysis confirmed that there was no significant relationship between drought (negative PDSI) and fire years (electronic supplementary material, table S5). We found that 36% of fire events occurred during warm and dry years, 50% during average years and 14% during cool and wet years (electronic supplementary material, figure S1). Fire years were not synchronous with years of significant departures from mean SSTs and SLP used as indexes for the ENSO, PDO and AO (electronic supplementary material, table S5 and figure S2). BEA analysis confirmed that antecedent drought (negative PDSI) values occurred more often than expected in the year preceding fire events (BEA, *p* = 0.005; figure 4*a*).

Positive phases of the ENSO (El Niño) occurred more often than expected 6–9 and 13–17 years prior to fire events (BEA, *p* = 0.005; figure 4*b*), and the positive phase of the PDO occurred more often than expected in the 10-year period prior to fire events (BEA, *p* = 0.005; figure 4*c*). Fires were consistently synchronous with the negative phase of the AO in the 33-year window prior to fire events (BEA, *p* = 0.001; figure 4*d*). Negative phases of the ENSO (La Niña) and the PDO were asynchronous with fire events and the positive phase of the AO was independent of fire events (electronic supplementary material, figure S3). To confirm that fire–climate relationships did not simply reflect signatures of natural climate variability, we performed BEA analysis on 16 randomly generated numbers (representing the 16 fire events) with non-significant results for all climate indexes (electronic supplementary material, figure S3).

### 3.4. Fire occurrence and interacting climate indexes

Fire frequency analyses of observed versus expected two- and three-way interactions between phase combinations of the ENSO, PDO and AO in the year of fire occurrence were non-significant (electronic supplementary material, table S5 and figure S2). This demonstrated that fire years were not more likely to occur during specific phase combinations and their associated climate signatures. Although fire and climate interactions did not follow an observable pattern in the year of fire events, interannual and multidecadal interactions between two- and three-way combinations of the ENSO, PDO and AO were significant (figure 4). Fires were synchronous with the combined positive phases of the ENSO and PDO 3–14 and 20–30 years prior to fire events (BEA, *p* = 0.005; figure 4*e*). Similarly, the negative phase of ENSO (La Niña) and the positive phase of the PDO occurred more often than expected 1–12 years prior to fire events (BEA, *p* = 0.001; figure 4*f*). Fires were consistently synchronous with the positive phase of the PDO and the negative phase of the AO during the 6–33-year period prior to fire events (BEA, *p* = 0.001; figure 4*g*). BEA analysis of interacting three-way phase combinations of the ENSO, PDO and AO were synchronous with fire events only when the ENSO and PDO were positive and the AO was negative 8–36 years prior to fire events (BEA, *p* = 0.001; figure 4*h*). All other phase combinations were non-significant (electronic supplementary material, figure S3).

## 4. Discussion

Drivers of historic fire activity remain largely unknown in coastal temperate rainforests in the PNW of North America [1,4,59]. We present the first examination of the influences of and interrelationships between humans and climate on fire activity in a very wet coastal temperate rainforest using a *ca* 700-year record of fire scars and stand establishment. Our data describe a low and mixed severity fire regime in which fires occurred on average every 39 years over the 517-year period from the first detected fire in 1376 to the last fire in 1893 (figure 2). Although the frequency of fire events on Hecate Island may seem low relative to fire-prone landscapes, our data indicate that fire occurrence was 25 times more likely than previous estimates of *ca* 1000-year intervals [14–16]. Such a fire frequency would have had large impacts on forest composition and structure, specifically in the AD 1600–1893 period when fires were more frequent (figure 2). The combination of low and mixed severity fires recurring at multidecadal intervals, the absence of fire after Indigenous abandonment (figure 2), and the spatial pattern of fire relative to habitation sites (figure 3) suggests that fire may have been intentionally used as a tool for resource management [9,60]. In addition, significant lagged effects of climate indexes prior to fire events indicate an added role of climate variability on fire occurrence (figure 4).

There is no evidence of fire activity after the last recorded fire in our study area in 1893 (figure 2). This 123-year fire-free interval (1893-present day) is distinct from the reconstructed fire history and is the longest fire-free period documented in the study area in seven centuries (figure 2). This period also coincides with the only time in roughly 13 000 years that the study area was not continuously inhabited by Indigenous peoples [31,32]. Unlike many regions of British Columbia that experienced widespread fire suppression in the twentieth century, there are no known records of ignitions or suppressions in this remote coastal temperate rainforest during the twentieth century, and very few in the entire coastal temperate rainforest as a whole [15,61]. However, several cycles of in-phase climate oscillations resulting in warmer and drier conditions conducive to fire spread occurred in the study area after the last recorded fire in 1893 (electronic supplementary material, table S1).

Fire–climate studies in the PNW relate synchronous fire years to the year's climate and cite warm and dry spring conditions [22], combined with low winter snowpack [7], as factors affecting fire years by drying fuels and resulting in longer fire seasons. Contrary to this, we found that antecedent drought in the year prior to fire events was significantly related to fire occurrence (figure 4*a*) and a closer examination of individual fire years revealed that fire events corresponded to 2–7 years of dry conditions (negative PDSI; electronic supplementary material, figure S1). The largest fire in the study area (287 ha) occurred in 1893, during one of the wettest (10th percentile) reconstructed years on record and one of the strongest negative ENSO (5th percentile) and PDO (25th percentile) climate years. Although the combined effects of these climate indexes suggest unsuitable conditions for fuel ignition and spread, a closer examination of the 10-year period prior to the fire year reveals a period of sustained drought and three of the strongest reconstructed positive ENSO (5th percentile) and PDO (10th percentile) years. This pattern of antecedent drought in combination with positive in-phase ENSO and PDO indexes prior to large fire events in the study area repeats through the dataset (figure 4) and reveals that antecedent drought may be a necessary precondition for fire initiation and spread at this study site. The effects of interannual and decadal drought on forest growth and fuel dynamics are not well documented in this region. Such antecedent climate events may affect the available fuel profile (as occurs in grassy fuel types such as in the southwest USA [24,62]) or other unknown aspects of the fire regime.

Our results are comparable to other studies in the PNW and indicate that interannual drought and interactions with regional climate variability may have been an important prerequisite for fuel combustion and fire spread [23], but our results are unique in concluding that fires would not likely have occurred without human ignitions (figure 3). For example, in our analyses, we found that fire events were not spatially biased to expected locations such as south-facing aspects [42], steep slopes [20] or rocky outcrops at middle and high elevations [19], but were strongly associated with the locations of former habitation sites (figure 3). We also found no difference in the frequency of fire events across the four vegetation types despite dissimilarities in fuel load, fuel availability and potential flammability (electronic supplementary material, figure 4) [21]. Although the effects of topography and vegetation on fire frequency were not readily apparent, the variables aspect and bog forest vegetation type were included in our final model selection (based on weighted probabilities) along with distance to former habitation site (table 1*b*). These variables may suggest other potential relationships between the spatial patterns of fire, vegetation and humans such as the influence of prevailing summer winds, natural fuel breaks and territorial boundaries. Despite being able to reconstruct historical fire events and gain insights into fire behaviour with the presence of fire-scarred trees [45], we could not confirm whether anthropogenic burning was accidental or intentional, or how often burning was attempted but unsuccessful because fire weather was not conducive to fuel ignition and fire spread [11].

Historically, low severity fires may have been intentionally set near habitation sites to clear land in otherwise dense forests, harvest timber and promote important and accessible food species such as berry-producing shrubs and bracken fern (*Pteridium aquilinium*), a fire follower species and an important starch in the diet of coastal Indigenous peoples [9,60]. In these very wet forests, centuries of repeat burning surrounding habitation sites may have affected fuel loads, seral stages and the abundance and distribution of flammable vegetation [1,2,24]. Because biomass is not limited in coastal temperate rainforests, humans can exert control over natural conditions that promote or reduce limits on fire activity. Therefore, human drivers and their ecosystem effects are probably more pronounced relative to long-term climate in these very wet ecosystems [1,2,4]. As climate change continues to promote prolonged warm and dry conditions and human footprints expand [8], fires in these ignition-limited systems may occur again, but have the potential to become large and uncontrollable [1,2,4,8]. We propose that a greater understanding of the cultural traditions of burning and their ecological effects would provide more insight into human controls on historic fire activity and help assess vulnerabilities to future human–fire–climate interactions [2].

## 5. Conclusion

Fires and their drivers have never been reconstructed with tree rings at centennial scales in coastal temperate rainforests. Although our study comprises only 300 ha in British Columbia, our results provide insight into the role of fire in very wet coastal temperate rainforests and the need to re-examine the fire regime concept to include human ignitions, fire as a tool for resource management and longer analysis periods [2]. Our results are consistent with other studies that suggest fire is rare in coastal temperate rainforest settings in the twentieth century [15–17], but we also found that historical fires occurred on average every 39 years during the AD 1376–1893 period and were synchronous with large-scale climate oscillations. Both Indigenous land use and climate variation appear to have interacted to create long-term trends in fire occurrence over the past 700 years, and we find the fire history of our study site is best explained by relating cycles of anthropogenic burning, which varied on decadal timescales, to decadal variability in climate. The ‘ecological legacies’ of historic anthropogenic burning, in the form of persistent effects on contemporary forest structure, probably abound in this region, and thus discredit the myth of coastal temperate rainforests as pristine landscapes. Our clear evidence of precontact anthropogenic burning calls into question our current understanding of fire history in coastal temperate rainforests and how we define landscapes that were historically managed with fire.

## Supplementary Material

1. Supporting tables, figures, and detailed methods for data analyses.

## Supplementary Material

2. Bivariate event analyses (BEA) used to compare tree-ring reconstructions of large-scale climate indices with fire events derived from fire-scarred trees.

## Supplementary Material

3. Tree-ring reconstructions of climate indices used in Bivariate Event Analyses (BEA) and 20th century records of average annual summer and winter temperature and precipitation.

## Supplementary Material

4. The supporting residual western redcedar chronology sampled from the study site and used in climate analyses

## Data accessibility

Datasets supporting this article have been uploaded as part of the electronic supplementary material.

## References

[1] McWethy DB et al. 2013 A conceptual framework for predicting temperate ecosystem sensitivity to human impacts on fire regimes. Glob. Ecol. Biogeogr. 22, 900–912. (doi:10.1111/geb.12038)

[2] Bowman DMJS et al. 2011 The human dimension of fire regimes on Earth. J. Biogeogr. 38, 2223–2236. (doi:10.1111/j.1365-2699.2011.02595.x) 22279247 10.1111/j.1365-2699.2011.02595.x PMC3263421

[3] Guyette RP, Muzika RM, Dey DC 2002 Dynamics of an anthropogenic fire regime. Ecosystems 5, 472–486. (doi:10.1007/s10021-002-0115-7)

[4] Whitman E, Batllori E, Parisien MA, Miller C, Coop JD, Krawchuk MA, Chong GW, Haire SL 2015 The climate space of fire regimes in north-western North America. J. Biogeogr. 42, 1736–1749. (doi:10.1111/jbi.12533)

[5] Foster DR, Clayden S, Orwig DA, Hall B, Barry S 2002 Oak, chestnut and fire: climatic and cultural controls of long-term forest dynamics in New England, USA. J. Biogeogr. 29, 1359–1379. (doi:10.1046/j.1365-2699.2002.00760.x)

[6] Lertzman K, Fall J 1998 From forest stands to landscapes: spatial scales and the roles of disturbances. In Ecological scale: theory and applications (eds DL Peterson, V Thomas Parker), pp. 339–367. New York, NY: Columbia University Press.

[7] Heyerdahl EK, Brubaker LB, Agee JK 2002 Annual and decadal climate forcing of historical fire regimes in the interior Pacific Northwest, USA. The Holocene 12, 597–604. (doi:10.1191/0959683602hl570rp)

[8] Ryan KC, Knapp EE, Varner MJ 2013 Prescribed fire in North American forests and woodlands: history, current practice, and challenges. Front. Ecol. Environ. 11, e15–e24. (doi:10.1890/120329)

[9] Turner NJ 2014 Ancient pathways, ancestral knowledge: ethnobotany and ecological wisdom of indigenous peoples of northwestern North America. Montreal, Quebec, Canada: McGill-Queen's Press-MQUP.

[10] Veblen TT, Kitzberger T, Donnegan J 2000 Climatic and human influences on fire regimes in ponderosa pine forests in the Colorado Front Range. Ecol. Appl. 10, 1178–1195. (doi:10.1890/1051-0761(2000)010[1178:CAHIOF]2.0.CO;2)

[11] Lepofsky D, Hallett D, Washbrook K, McHalsie A, Lertzman K, Mathewes R 2005 Documenting precontact plant management on the Northwest Coast: an example of prescribed burning in the central and upper Fraser Valley, British Columbia. In Keeping it living: traditions of plant use and cultivation on the Northwest Coast, pp. 218–239.

[12] Gedalof Z, Pellatt M, Smith DJ 2006 From prairie to forest: three centuries of environmental change at Rocky Point, Vancouver Island, British Columbia. Northwest Sci. 80, 34–46.

[13] Agee JK 1996 Fire ecology of Pacific Northwest forests. Washington, DC: Island Press.

[14] Daniels LD, Gray RW 2006 Disturbance regimes in coastal British Columbia. J. Ecosyst. Manag. 7, 44–56.

[15] Wong C, Dorner B, Sandmann H 2003 Estimating historical variability of natural disturbances in British Columbia. Victoria, Canada: Ministry of Forests Research Branch.

[16] Pearson AF 2010 Natural and logging disturbances in the temperate rain forests of the central coast, British Columbia. Can. J. For. Res. 40, 1970–1984. (doi:10.1139/X10-137)

[17] Meyn A, Taylor SW, Flannigan MD, Thonicke K, Cramer W 2010 Relationship between fire, climate oscillations, and drought in British Columbia, Canada, 1920–2000. Glb. chg. bio. 16, 977–989. (doi:10.1111/j.1365-2486.2009.02061.x)

[18] Brown K, Hebda RJ 2002 Origin, development, and dynamics of coastal temperate conifer rainforests of southern Vancouver Island, Canada. Can. J. For. Res. 32, 353–372. (doi:10.1139/x01-197)

[19] Lertzman K, Gavin D, Hallett D, Brubaker L, Lepofsky D, Mathewes R 2002 Long-term fire regime estimated from soil charcoal in coastal temperate rainforests. Conserv. Ecol. 6, 5.

[20] Gavin DG, Brubaker LB, Lertzman KP 2003 Holocene fire history of a coastal temperate rain forest based on soil charcoal radiocarbon dates. Ecology 84, 186–201. (doi:10.1890/0012-9658(2003)084[0186:HFHOAC]2.0.CO;2)

[21] Hoffman KM, Gavin DG, Lertzman KP, Smith DJ, Starzomski BM 2016 13,000 years of fire history derived from soil charcoal in a British Columbia coastal temperate rainforest. Ecosphere 7, e01415 (doi:10.1002/ecs2.1415)

[22] Hessl AE, McKenzie D, Schellhaas R 2004 Drought and Pacific Decadal Oscillation linked to fire occurrence in the inland Pacific Northwest. Ecol. Appl. 14, 425–442. (doi:10.1890/03-5019)

[23] Gedalof Z, Peterson DL, Mantua NJ 2005 Atmospheric, climatic, and ecological controls on extreme wildfire years in the northwestern United States. Ecol. Appl. 15, 154–174. (doi:10.1890/03-5116)

[24] Kitzberger T, Brown PM, Heyerdahl EK, Swetnam TW, Veblen TT 2007 Contingent Pacific–Atlantic Ocean influence on multicentury wildfire synchrony over western North America. Proc. Natl Acad. Sci. USA 104, 543–548. (doi:10.1073/pnas.0606078104) 17197425 10.1073/pnas.0606078104 PMC1766421

[25] D'Arrigo R, Cook ER, Wilson RJ, Allan R, Mann ME 2005 On the variability of ENSO over the past six centuries. Geophys. Res. Lett. 32, L03711 (doi:10.1029/2004GL022055)

[26] Mantua NJ, Hare SR 2002 The Pacific decadal oscillation. J. Oceanogr. 58, 35–44. (doi:10.1023/A:1015820616384)

[27] Thompson DW, Wallace JM 2000 Annular modes in the extratropical circulation. Part I: month-to- month variability. J. Clim. 13, 1000–1016. (doi:10.1175/1520-0442(2000)013<1000:AMITEC>2.0.CO;2)

[28] Thompson DW, Wallace JM 1998 The Arctic Oscillation signature in the wintertime geopotential height and temperature fields. Geophys. Res. Lett. 25, 1297–1300. (doi:10.1029/98GL00950)

[29] Macias Fauria M, Johnson EA 2006 Large-scale climatic patterns control large lightning fire occurrence in Canada and Alaska forest regions. J. Geophys. Res. 111, G04008 (doi:10.1029/2006JG000181)

[30] Skinner WR, Flannigan MD, Stocks BJ, Martell DL, Wotton BM, Todd JB, Mason JA, Logan KA, Bosch EM 2002 A 500 hPa synoptic wildland fire climatology for large Canadian forest fires, 1959–1996. Theor. Appl. Climatol. 71, 157–169. (doi:10.1007/s007040200002)

[31] McLaren D, Fedje D, Hay MB, Mackie Q, Walker IJ, Shugar DH, Eamer JB, Lian OB, Neudorf C 2014 A post-glacial sea level hinge on the central Pacific coast of Canada. Quat. Sci. Rev. 97, 148–169. (doi:10.1016/j.quascirev.2014.05.023)

[32] McLaren D, Rahemtulla F, Fedje D 2015 Prerogatives, sea level, and the strength of persistent places: archaeological evidence for long-term occupation of the Central Coast of British Columbia. BC Stud. Brit. Columb. Q. 187, 155–191.

[33] Banner A, LePage P, Moran J, de Groot A 2005 The HyP3 project: pattern, process, and productivity in hypermaritime forests of coastal British Columbia: a synthesis of 7-year results. Victoria, Canada: Ministry of Forests Research Branch.

[34] Meidinger DV, Pojar J 1991 Ecosystems of British Columbia. Victoria, Canada: Special Report Series-Ministry of Forests.

[35] Klinka K, Qian H, Pojar J, Meidinger DV 1996 Classification of natural forest communities of coastal British Columbia, Canada. Vegetatio 125, 149–168. (doi:10.1007/BF00044648)

[36] Roddick JA 1996 Queens Sound (102P) British Columbia: Geological Survey of Canada, Open File 3278, scale 1:250,000. Ottawa, Canada: Natural Resources Canada.

[37] Trant AJ, Nijland W, Hoffman KM, McLaren D, Mathews DL, Nelson TA, Starzomski BM 2016 Intertidal resource use over millennia enhances forest productivity. Nat. Commun. 7, 12491 (doi:10.1038/ncomms12491) 27572157 10.1038/ncomms12491 PMC5013557

[38] Boyd R 1994 Smallpox in the Pacific Northwest: the first epidemics. BC Stud. 101, 5–40.

[39] Olson RL 1955 Notes on the Bella Bella Kwakiutl, vol. 14. Berkeley, CA: University of California Press.

[40] Arno SF, Sneck KM 1977 A method for determining fire history in coniferous forests of the mountain west. Ogden, UT: Intermountain Forest and Range Experiment Station, US Department of Agriculture.

[41] Johnson EA, Gutsell SL 1994 Fire frequency models, methods and interpretations. Adv. Ecol. Res. 25, 153–166.

[42] Heyerdahl EK, Brubaker LB, Agee JK 2001 Spatial controls of historical fire regimes: a multiscale example from the interior west, USA. Ecology 82, 660–678. (doi:10.1890/0012-9658(2001)082[0660:SCOHFR]2.0.CO;2)

[43] Stokes MA, Smiley TL 1968 An introduction to tree-ring dating. Chicago, IL: University of Chicago Press.

[44] Grissino-Mayer HD 2001 Evaluating crossdating accuracy: a manual and tutorial for the computer program COFECHA. Tree-ring Res. 57, 205–221.

[45] Falk DA, Heyerdahl EK, Brown PM, Farris C, Fulé PZ, McKenzie D, Swetnam TW, Taylor AH, Van Horne ML 2011 Multi-scale controls of historical forest-fire regimes: new insights from fire-scar networks. Front. Ecol. Environ. 9, 446–454. (doi:10.1890/100052)

[46] Brewer PW, Velásquez ME, Sutherland EK, Falk DA 2015 Fire History Analysis and Exploration System (FHAES) version 2.0.0 [computer software]. See http://www fhaes.org.

[47] Barton K 2016 MuMIn: Multi-model inference. R package version 1.10. 14.

[48] R Development Core Team. 2016 R: a language and environment for statistical computing, reference index version 3.0. Vienna, Austria: R Foundation for Statistical Computing.

[49] Harrell F 2015 Regression modeling strategies: with applications to linear models, logistic and ordinal regression, and survival analysis. Berlin, Germany: Springer.

[50] Burnham K, Anderson D 2002 Model selection and multi-model inference. Berlin, Germany: Springer.

[51] Palmer WC 1965 Meteorological drought. Washington, DC: US Department of Commerce, Weather Bureau.

[52] Cook ER, Krusic PJ 2004 North American summer PDSI reconstructions. IGBP PAGES/World Data Center for Paleoclimatology Data Contribution Series 45.

[53] Signorell A et al. 2015 DescTools: Tools for descriptive statistics. R package version 0.99 15.

[54] Schoennagel T, Veblen TT, Kulakowski D, Holz A 2007 Multidecadal climate variability and climate interactions affect subalpine fire occurrence, western Colorado (USA). Ecology 88, 2891–2902. (doi:10.1890/06-1860.1) 18051658 10.1890/06-1860.1

[55] Gavin DG 2010 K1D: Multivariate Ripley's K-function for one-dimensional data. [computer software]. See http://geography.uoregon.edu/envchange/pbl/software.html.

[56] Gavin DG, Hu FS, Lertzman K, Corbett P 2006 Weak climatic control of stand-scale fire history during the late Holocene. Ecology 87, 1722–1732. (doi:10.1890/0012-9658(2006)87[1722:WCCOSF]2.0.CO;2) 16922322 10.1890/0012-9658(2006)87[1722:wccosf]2.0.co;2

[57] Carcaillet C, Ali AA, Blarquez O, Genries A, Mourier B, Bremond L 2009 Spatial variability of fire history in subalpine forests: from natural to cultural regimes. Ecoscience 16, 1–12. (doi:10.2980/16-1-3189)

[58] Bigler C, Gavin DG, Gunning C, Veblen TT 2007 Drought induces lagged tree mortality in a subalpine forest in the Rocky Mountains. Oikos 116, 1983–1994. (doi:10.1111/j.2007.0030-1299.16034.x)

[59] Whitlock C, Higuera PE, McWethy DB, Briles CE 2010 Paleoecological perspectives on fire ecology: revisiting the fire-regime concept. Open Ecol. J. 3, 6–23. (doi:10.2174/1874213001003020006)

[60] Turner NJ 1999 ‘Time to burn:’ traditional use of fire to enhance resource production by Aboriginal Peoples in British Columbia. In Indians, Fire and the Land in the Pacific Northwest (ed. R Boyd), pp. 185–218. Corvallis, OR: Oregon State University Press.

[61] Stocks BJ et al. 2002 Large forest fires in Canada, 1959–1997. J. Geophys. Res. 107, 8149 (doi:10.1029/2001JD000484)

[62] Swetnam TW, Betancourt JL 1998 Mesoscale disturbance and ecological response to decadal climatic variability in the American Southwest. J. Clim. 12, 3128–3147. (doi:10.1175/1520-0442(1998)011<3128:MDAERT>2.0.CO;2)

