# Supplementary material for: Seven hundred years of human-driven and climate-influenced fire activity in a British Columbia coastal temperate rainforest
Source: R Soc Open Sci. 2016 Oct 26;3(10):160608. doi: 10.1098/rsos.160608 (PMC5099006; doi:10.1098/rsos.160608)

Electronic Supplementary Material

Table S1: Tree-ring reconstructed climate chronologies used in analyses of fire activity on Hecate Island, British Columbia, Canada. Abbreviations are the El Niño-Southern Oscillation (ENSO), the Pacific Decadal Oscillation (PDO), the Palmer Drought Severity Index (PDSI), and the Arctic Oscillation (AO). Note correlations with instrumental record of temperature are in summer months unless indicated by (DJF [December, January, and February]). Statistically significant relationships are indicated with (*). The supporting residual western redcedar chronology is provided in the additional supplementary material.

Climate index and time period (AD)	Season reconstructed	% variance explained in the instrumental record	Correlation with instrumental record of temperature in the study area (AD 1900-1975)	Correlation with residual tree-ring chronology in the study area (AD 1330-2014)
ENSO[1] (AD 1300-2006)	Dec.-Feb.	51% (AD 1870-2002)	0.40 $P < 0.001$*	0.28 $P < 0.05$*
PDO[2] (AD 1700-1997)	Dec.-Feb.	53% (AD 1900-1979)	0.34 $P < 0.001$*	0.28 $P < 0.01$*
PDSI[3] (AD 1249-2004)	June-Aug.	40% (AD 1928-1978)	-0.28 (DJF) $P < 0.001$*	-0.21 $P < 0.05$*
AO[4] (AD 1650-1978)	April-Sep.	48% (AD 1900-1975)	-0.33 (DJF) $P < 0.005$*	-0.19 $P = 0.10$

Note: All reconstructions were downloaded from National Climatic Data Center, National Oceanic and Atmospheric Administration and are freely available (http://hurricane. ncdc. noaa. gov/pls/paleox/)

References:

[1] Li J. *et al*. 2013 El Niño modulations over the past seven centuries. *Nature Climate Change* **3**(9), 822–826.
[2] D'Arrigo RD, Villalba R. and G Wiles. 2001 Tree-ring estimates of Pacific decadal climate variability. *Climate Dynamics* **18**, 219–224.
[3] Cook ER and Krusic PJ. 2004 North American summer PDSI reconstructions. *IGBP PAGES/World Data Center for Paleoclimatology Data Contribution Series* **45**.
[4] D'Arrigo RD, Cook ER, Mann ME and Jacoby GC. 2003 Tree-ring reconstructions of temperature and sea-level pressure variability associated with the warm-season Arctic Oscillation since AD 1650. *Geophysical Research Letters* **30**.

Table S2: Summary of the fire scar and stand establishment data used to reconstruct the fire history of 30 plots on Hecate Island, British Columbia, Canada. Data from each plot include: the vegetation type (ZF = zonal forest, BF = bog forest, BW = bog woodland and BB = blanket bog), the location (latitude and longitude), the number of fire scars, the number of years recording more than two fire scars, the year of the fire and the length in years of the fire scar record. The age range and post-fire recruitment decade of trees and the point fire interval (PFI [the mean fire interval at the tree scale]) for each plot are provided.

Type	Coordinates Lat/Long	# of fire scars	# of yrs with > 2 scars	First fire scar yr	Last fire scar yr	Fire scar event yrs	Stand age range (yrs) and AD distribution	Post-fire cohort (decade)	Point fire interval (yrs)
ZF	51°39'50.7"N 128°4'36.1"W	14	4	1376	1893	1376, 1482, 1537, 1699, 1744, 1759, 1778, 1797, 1893	54-380 (1634-2014)	1760, 1770, 1830	47
ZF	51°39'48.8"N 128° 4'43.5"W	2	0	1778	1893	1778, 1893	95-345 (1669-2014)	1660, 1740, 1770, 1880	103
ZF	51°39'34.9"N 128° 4'36.6"W	1	0	1593	1893	1593, 1778, 1893	83-493 (1521-2014)	1750, 1800, 1890	103
ZF	51°39'35.8"N 128° 4'10.7"W	21	4	1656	1893	1656, 1744, 1759, 1856, 1893	79-496 (1518-2014)	1740, 1760, 1890	73
ZF	51°40'1.4"N 128° 4'27.9"W	3	2	1744	1893	1744, 1759, 1844, 1893	115-620 (1394-2014)	1670, 1740, 1790, 1810	86
BF	51°39'59.7"N 128° 4'4.8"W	7	3	1744	1893	1744, 1759, 1778, 1848, 1893	84-464 (1550-2014)	1700, 1850, 1880	73
BF	51°39'59.7"N 128° 4'14.5"W	3	2	1744	1893	1744, 1759, 1778, 1893	85-563 (1451-2014)	1850	103
BF	51°40'0.3"N 128° 3'50.9"W	6	3	1719	1893	1719, 1744, 1759, 1778, 1893	71-414 (1600-2014)	1670, 1890, 1900	73
BF	51°39'56.6"N 128° 3'42.1"W	6	4	1719	1893	1719, 1744, 1759, 1778, 1893	73-445 (1569-2014)	1890, 1900	103
BF	51°39'54.3"N 128° 3'57.4"W	5	5	1656	1893	1656, 1744, 1759, 1778, 1893	63-533 (1481-2014)	1720, 1900	73
BF	51°39'49.5"N 128° 4'32.9"W	25	7	1376	1893	1376, 1482, 1537, 1699, 1744, 1759,	112-443 (1571-2014)	1800, 1890	51

						1778, 1797, 1893			
BF	51°39'47.2"N 128° 4'38.4"W	21	5	1537	1893	1537, 1656, 1744, 1778, 1797, 1893	92-350 (1664-2014)	1790, 1890	86
BF	51°39'37.8"N 128° 5'2.8"W	5	2	1778	1893	1778, 1893	52-579 (1435-2014)	1630, 1720, 1900	129
BF	51°39'31.0"N 128° 3'4.8"W	0	0	NA	NA	No fire scar evidence	120-953 (1061-2014)	1790, 1840	No fire evidenc e
BW	51°39'56.5"N 128° 4'25.3"W	6	5	1656	1893	1656, 1744, 1759, 1778, 1893	72-463 (1551-2014)	1800, 1890	86
BW	51°40'11.1"N 128° 4'15.2"W	0	0	1893	NA	1893	118-361 (1653-2014)	1800, 1860, 1890	172
BW	51°39'52.7"N 128° 3'52.0"W	6	3	1744	1893	1744, 1759, 1778, 1893	52-570 (1441-2014)	1890	129
BW	51°39'39.1"N 128° 4'19.5"W	18	6	1656	1893	1656, 1744, 1759, 1778, 1856 1893	59-531 (1483-2014)	1890	73
BW	51°39'52.1"N 128° 3'25.9"W	0	0	1893	NA	1893	72-310 (1704-2014)	1890, 1900	1 fire event
BW	51°39'46.7"N 128° 4'2.7"W	5	3	1656	1893	1656, 1744, 1759, 1778, 1893	82-119 (1895-2014)	1890, 1900	103
BW	51°39'38.3"N 128° 4'30.3"W	15	3	1656	1893	1656, 1744, 1778, 1797, 1856, 1893	59-311 (1703-2014)	1900	86
BW	51°39'37.7"N 128° 3'19.1"W	0	0	1893	NA	1893	70-360 (1654-2014)	1900	1 fire event
BW	51°39'34.0"N 128° 3'48.0"W	3	0	1893	NA	1893	76-293 (1721-2014)	NA	1 fire event
BW	51°39'31.8"N 128° 4'47.5"W	2	1	1893	NA	1893	49-533 (1481-2014)	1890, 1900	1 fire event
BB	51°39'59.7"N 128° 3'27.0"W	0	0	1893	NA	1893	71-171 (1843-2014)	1890	1 fire event
BB	51°39'42.0"N 128° 4'12.7"W	26	10	1656	1893	1656, 1744, 1759, 1778, 1893	52-120 (1894-2014)	NA	103
BB	51°39'46.1"N 128° 3'34.9"W	1	0	1893	NA	1893	57-120 (1894-2014)	1900	1 fire event

BB	51°39'33.1"N 128° 3'56.8"W	6	0	1893	NA	1893	74-110 (1904-2014)	NA	1 fire event
BB	51°40'11.2"N 128° 3'36.1"W	1	0	1893	NA	1778, 1893	120-163 (1851-2014)	1780	258
BB	51°39'49.1"N 128° 4'14.6"W	4	2	1656	1893	1656, 1744, 1759, 1778, 1893	91-219 (1705-2014)	1890	103

Table S3-S4: Candidate Poisson GLM models were selected with Akaike Information Criterion (AIC_c) for the two response variables: 1) abundance of fire-scarred trees, and; 2) frequency of fire events with nine predictor variables (vegetation type [four types], elevation, slope, aspect, distance to habitation site in meters, and distance to shoreline in meters). Model averaging was conducted and the most parsimonious model related the abundance of fire scars to distance from former habitation sites only and this was confirmed with model validation of residuals. In the second analysis, the most parsimonious model related the frequency of fire events to three variables (distance from former habitation site, aspect and bog forest vegetation type). The explained deviance (*pseudo* R^2) is the quality of fit achieved by maximum likelihood for the model.

**S3. Abundance of fire-scarred trees**					
**Step 1:** Assess collinearity in predictor variables	All variables have Variance Inflation Factor (VIF < 3), no evidence of correlation among variables				
**Step 2**: Specify parameters (variables) to include in the model	Elevation, slope, aspect, distance to habitation, distance to shoreline, vegetation type (zonal forest, bog forest, bog woodland and blanket bog)				
**Step 3**: Choose an appropriate distribution	Poisson distribution for count data				
**Step 4:** Graphically check the variances in the data, outliers, etc.	No apparent issues and no assumptions violated				
**Step 5:** Fit the beyond optimal (global) GLM and undertake model selection with AIC_c	Elevation, slope, aspect, distance to habitation, distance to shoreline and vegetation type. AIC_c 126.0. Model uncertainty exists; there are several models within the 95% confidence set of models. Model averaging is warranted. Note that vegetation type was not a parameter in the 95% confidence interval set of models.				
**Step 6**: Apply model averaging and 95% confidence intervals	**Parameter**	**Estimate**	**Unconditional Standard Error**	**95% Confidence intervals**	**Relative importance**
	Intercept	1.963e+00	1.707e-01	(1.591, 2.372)	
	Elevation	-4.302e-03	6.790e-03	(-0.0178, 0.009)	0.15
	Distance to shoreline	2.458e-04	8.440e-04	(-0.001, -0.001)	0.13
	Distance to habitation	-2.002e-03	4.383e-04	(-0.002, -0.001)	1.00
	Slope	-1.114e-02	1.350e-02	(-0.038, 0.0166)	0.17
	Aspect	3.255e-05	9.327e-04	(-0.002, 2.508)	0.12
**Step 7**: Select the most parsimonious model from the selection with AIC_c	**Parameters**	**Log-Likelihood**	**AIC_c**	**Delta**	**Weight**
	Distance to habitation	-51.81	108.06	0.00	0.42
	Distance to habitation, slope	-51.45	109.83	1.77	0.17

	Distance to habitation, elevation	-51.58	110.08	2.02	0.15
	Distance to habitation, aspect	-51.76	110.44	2.39	0.13
**Step 8:** Select the most parsimonious model	The most parsimonious model contained the parameter distance to habitation only and was the most significant predictor.				
**Step 9:** Assess model adequacy	We used several graphical methods to assess model adequacy and found no evidence of overdispersion or departures from model assumptions. Distance from habitation explained 65% of the variation in the abundance of fire-scarred trees (*pseudo* R^2).				
**S4. Frequency of fire events**					
**Step 1:** Assess collinearity in predictor variables	All variables have Variance Inflation Factor (VIF < 3), no evidence of correlation among variables				
**Step 2**: Specify parameters (variables) to include in the model	Elevation, slope, aspect, distance to habitation, distance to shoreline, vegetation type (zonal forest, bog forest, bog woodland and blanket bog)				
**Step 3**: Choose an appropriate distribution	Poisson distribution for count data				
**Step 4:** Graphically check the variances in the data, outliers, etc.	No apparent issues and no assumptions violated				
**Step 5:** Fit the beyond optimal (global) GLM and undertake model selection with AIC_c	Elevation, slope, aspect, distance to habitation, distance to shoreline and vegetation type. AIC_c 119.3. Model uncertainty exists; there are several models within the 95% confidence set of models. Model averaging is warranted.				
**Step 6**: Apply model averaging and 95% confidence intervals	**Parameter**	**Estimate**	**Unconditional Standard Error**	**95% Confidence intervals**	**Relative importance**
	Intercept	1.678	0.270	(1.094, 2.244)	
	Elevation	-0.003	0.006	(-1.664, 0.125)	0.15
	Distance to shoreline	0.001	0.0007	(1.971, 0.002)	0.50
	Distance to habitation	-0.002	0.0006	(-3.532, -0.001)	1.00
	Slope	0.002	0.011	(-2.657, 0.026)	0.18
	Aspect	0.001	0.0008	(-0.002, 2.508)	0.76
	Bog forest	0.716	0.317	(-6.479, 1.315)	0.54
	Bog woodland	0.030	0.365	(-7.084,0.792)	0.44
	Zonal forest	0.367	0.366	(-3.633, 1.130)	0.52
	Blanket bog	0.425	0.331	(-1.002, 0.013)	0.44
**Step 7**: Select the most parsimonious	**Parameters**	**Log-Likelihood**	**AIC_c**	**Delta**	**Weight**

model from the selection with AIC_c	Distance to habitation, aspect, bog forest type	-56.31	119.54	0.00	0.30
	Distance to habitation, aspect	-52.32	120.29	0.75	0.20
	Distance to habitation	-54.67	121.85	2.31	0.09
	Distance to habitation, aspect, bog forest type, zonal forest type, distance to shoreline	-56.23	122.07	2.53	0.08
	Distance to habitation, elevation, slope, distance to shoreline	-56.30	122.21	2.67	0.08
	Distance to habitation, bog forest type, slope	-58.93	122.30	2.76	0.07
	Distance to habitation, elevation, bog forest type	-52.01	123.12	3.58	0.05
**Step 8:** Select the most parsimonious model	The most parsimonious model contained the parameters distance to habitation, aspect, and bog forest type. Distance to habitation site is the most important predictor variable followed by aspect and bog forest vegetation type.				
**Step 9:** Assess model adequacy	We used a range of graphical methods to assess model adequacy (see Appendix S4) and we found no evidence of overdispersion or departures from model assumptions. Distance from habitation, aspect, and bog forest vegetation type explained 59% of the variation in the frequency of fire events (*pseudo* R^2).				

Table S5: G-test of goodness of fit of observed versus expected frequencies of fire occurrence and single and multiple phase interactions of the PDSI, ENSO, PDO and AO climate indexes. Abbreviations are the El Niño-Southern Oscillation (ENSO), the Pacific Decadal Oscillation (PDO), the Palmer Drought Severity Index (PDSI) and the Arctic Oscillation (AO). All single and phase combinations were nonsignificant.

**Climate Index**	**G-test of goodness of fit**	**X^2 Degrees of freedom**	***P*-value**
PDSI	$G = 3.1916$	1	0.07402
ENSO	$G = 0.5576$	1	0.4552
PDO	$G = 0.603$	1	0.4374
AO	$G = 0.6254$	1	0.4291
ENSO x PDO	$G = 0.3286$	3	0.9546
ENSO x AO	$G = 1.714$	3	0.6338
PDO x AO	$G = 1.686$	3	0.64
ENSO x PDO x AO	$G = 3.2029$	7	0.8656

Figure S1: Years of fire occurrence in the study area on Hecate Island, British Columbia, Canada are indicated with red diamond symbols. Tree-ring reconstructions of the Palmer Drought Severity Index (PDSI (green), El Niño-Southern Oscillation (ENSO) (orange), Pacific Decadal Oscillation (PDO) (blue), and the Arctic Oscillation (AO) (pink) indexes are plotted with their associated warm/dry and cool/wet phases during the analysis period AD 1300-1900. References for tree-ring reconstructions are included in the electronic supplementary material, table S1.

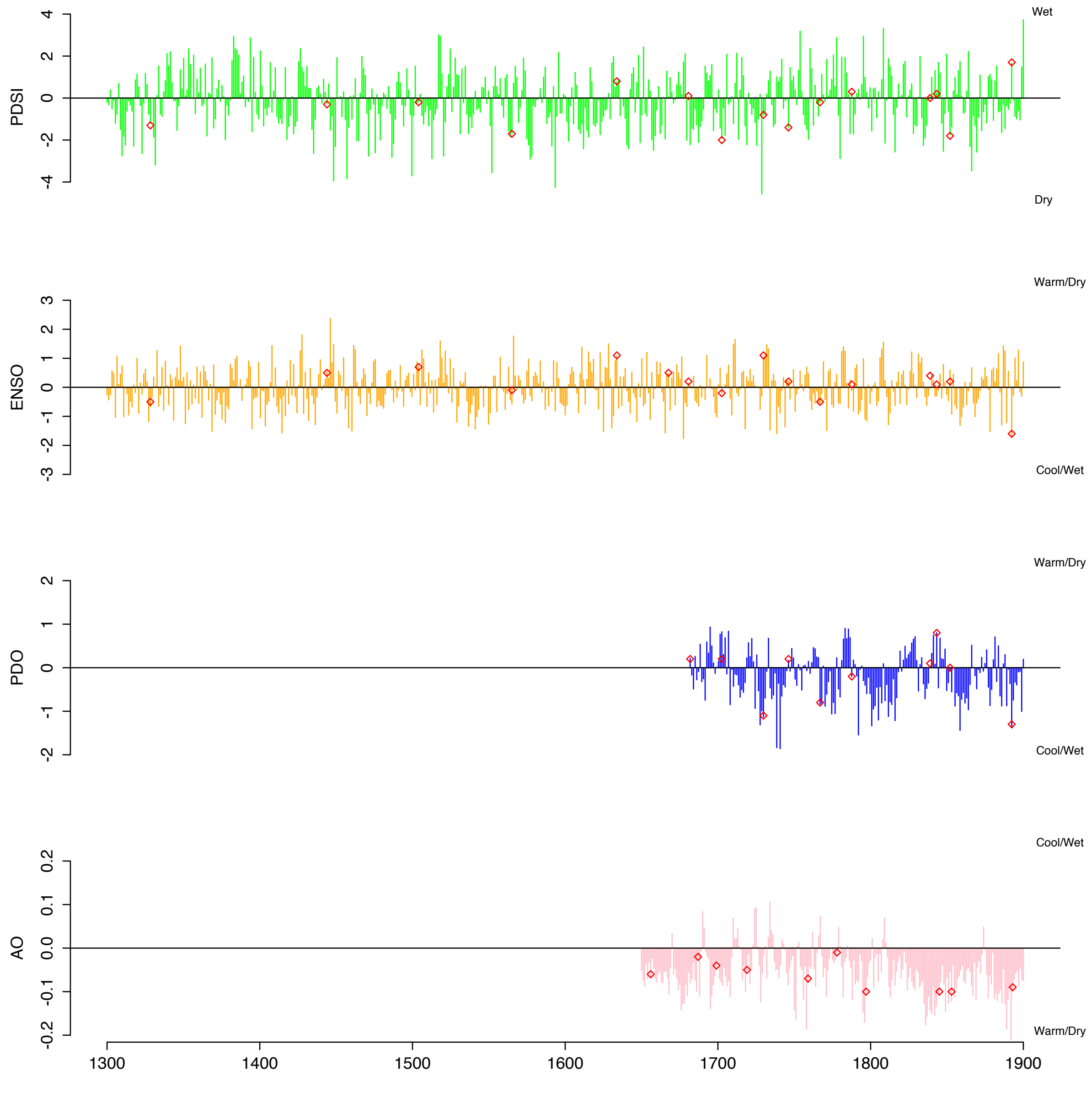

Figure S2: Relative frequencies of observed (black bars) versus expected (grey bars) fire events and climate occurrences for each of the two-way (a-c) and three-way (d) combined phases of the El Niño-Southern Oscillation (ENSO), Pacific Decadal Oscillation (PDO) and Arctic Oscillation (AO) climate indexes (see methods: single index and multiple index combinations). Positive phases are indicated with the (+) symbols and negative phases with the (–) symbols. Significant departures from expected fire occurrence were evaluated with the G-test of goodness of fit for all single, two-way, and three-way interactions.

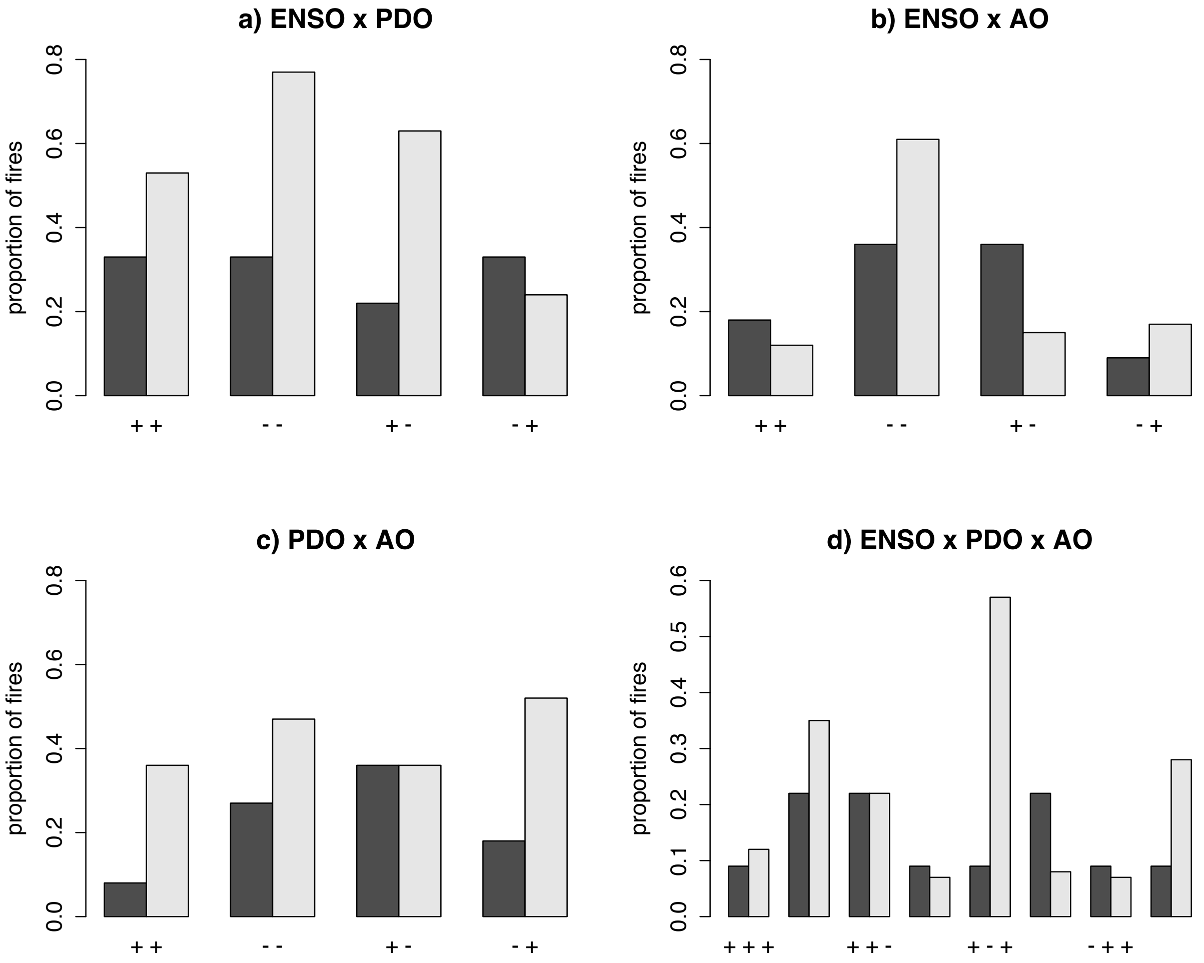

Figure S3: Fifteen Bivariate Event Analyses (BEA) of the temporal associations between fire years (n = 16) and extreme climate events (single indexes n = 50, combined indices n = 100). Black lines above the dotted red (99% confidence envelopes) and the dotted grey (95% confidence envelopes) lines indicate synchrony between the two events (events occurred more often than expected, *t* years prior to fire events) and black lines below lower confidence envelopes indicate asynchrony (extreme events occur less often than expected *t* years prior to fire events). Black lines between confidence envelopes indicate independence between events. Confidence envelopes are based on 1000 Monte Carlo simulations and years of significant asynchrony are shaded in grey.

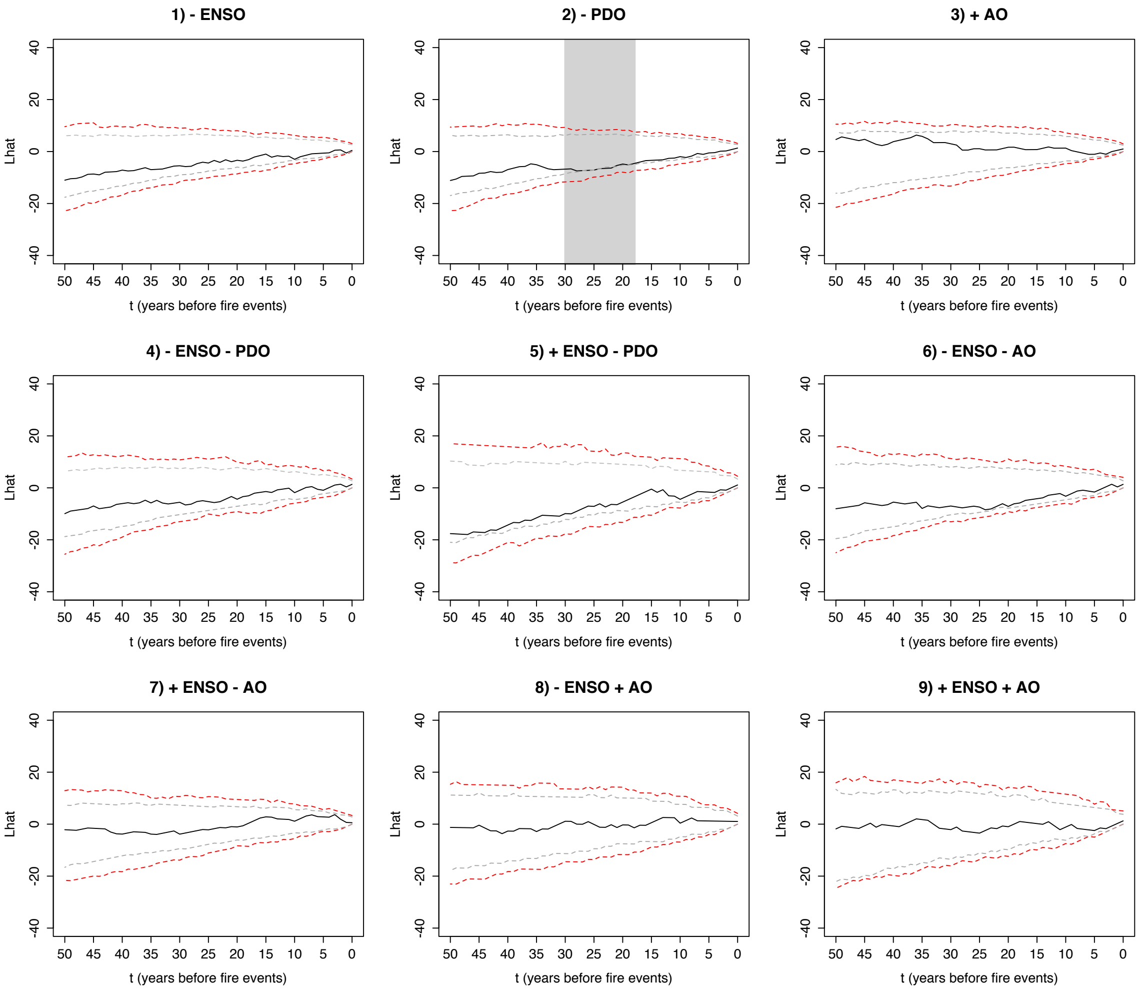

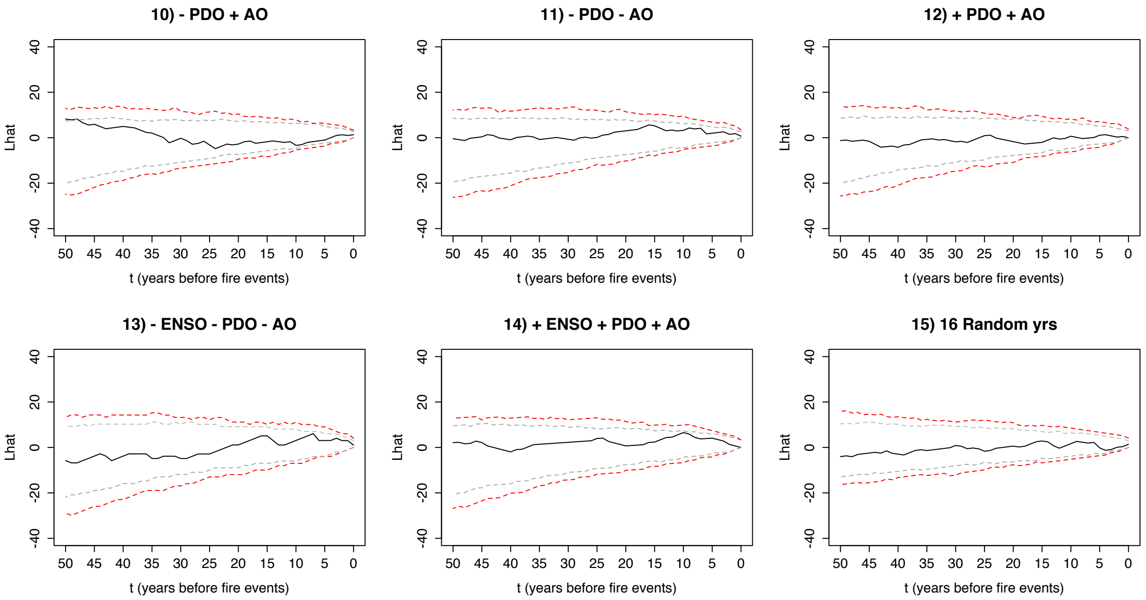
10) - PDO + AO
11) - PDO - AO
12) + PDO + AO
13) - ENSO - PDO - AO
14) + ENSO + PDO + AO
15) 16 Random yrs
Lhat
t (years before fire events)
40
20
0
-20
-40
50 45 40 35 30 25 20 15 10 5 0

Figure S4: Point fire intervals (PFI [mean fire interval obtained from all trees within one-hectare plots]) were pooled by vegetation type (four types differentiated by different shades of green) during the analysis period AD 1376-1893. The number of plots assessed is given in parentheses. The boxes enclose the 25th to 75th percentiles, and whiskers enclose the 10th to 90th percentiles. The horizontal lines across each box indicate the median. No pairwise comparisons in median PFI were statistically significant.

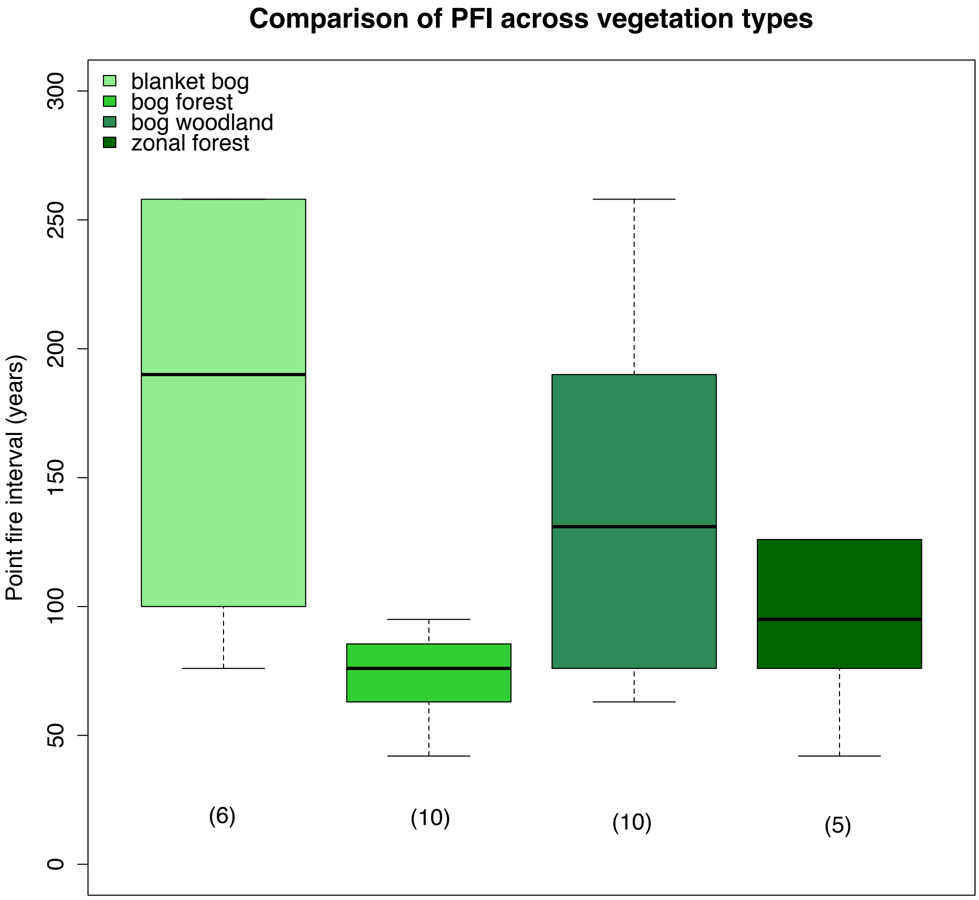

Supplement: 1. Supporting tables, figures, and detailed methods for data analyses. [file rsos160608supp1.pdf]
